# Efficacy of azilsartan on left ventricular diastolic dysfunction compared with candesartan: J-TASTE randomized controlled trial

**DOI:** 10.1038/s41598-023-39779-y

**Published:** 2023-08-02

**Authors:** Shin Ito, Hiroyuki Takahama, Masanori Asakura, Yukio Abe, Masayoshi Ajioka, Toshihisa Anzai, Takuo Arikawa, Takaharu Hayashi, Yorihiko Higashino, Shinya Hiramitsu, Noriaki Iwahashi, Chisato Izumi, Kazuo Kimura, Koichiro Kinugawa, Hidetaka Kioka, Young-Jae Lim, Ken Matsuoka, Satoshi Matsuoka, Hirohiko Motoki, Sunao Nakamura, Takafumi Nakayama, Akihiro Nomura, Taishi Sasaoka, Shin Takiuchi, Shigeru Toyoda, Tomoya Ueda, Tetsuya Watanabe, Akira Yamada, Masayoshi Yamamoto, Takashi Sozu, Masafumi Kitakaze

**Affiliations:** 1 Department of Cardiovascular Medicine, National Cerebral and Cardiovascular Center, Osaka, Japan; 2 Department of Clinical Medicine and Development, National Cerebral and Cardiovascular Center, Osaka, Japan; 3 Department of Cardiovascular Medicine, Tohoku University Graduate School of Medicine, Sendai, Japan; 4 Department of Cardiovascular and Renal Medicine, Hyogo College of Medicine, Nishinomiya, Japan; 5 Department of Cardiology, Osaka City General Hospital, Osaka, Japan; 6 Department of Cardiovascular Internal Medicine, Tosei General Hospital, Seto, Aichi Japan; 7 Department of Cardiovascular Medicine, Hokkaido University Graduate School of Medicine, Sapporo, Hokkaido Japan; 8 Department of Cardiovascular Medicine, Dokkyo Medical University, Mibu, Tochigi Japan; 9 Cardiovascular Medicine, Osaka Police Hospital, Osaka, Japan; 10 Department of Cardiology, Higashi Takarazuka Satoh Hospital, Takarazuka, Hyogo Japan; 11 Cardiology, Hiramitsu Heart Clinic, Nagoya, Japan; 12 Division of Cardiology, Yokohama City University Medical Center, Yokohama, Japan; 13 The Second Department of Internal Medicine, University of Toyama, Toyama, Japan; 14 Department of Cardiovascular Medicine, Osaka University Graduate School of Medicine, Osaka, Japan; 15 Cardiovascular Center, Kawachi General Hospital, Osaka, Japan; 16 Department of Internal Medicine, Yoshikawa Hospital, Osaka, Japan; 17 Department of Cardiology, New Tokyo Hospital, Chiba, Japan; 18 Department of Cardiovascular Medicine, Shinshu University School of Medicine, Nagano, Japan; 19 Department of Cardiology, Nagoya City University Graduate School of Medical Sciences, Nagoya, Aichi Japan; 20 Innovative Clinical Research Center/Department of Cardiovascular Medicine, Kanazawa University Graduate School of Medical Sciences, Kanazawa, Japan; 21 Internal Medicine, Watanabe Clinic, Saitama, Japan; 22 Department of Cardiovascular Medicine, Nara Medical University, Nara, Japan; 23 Division of Cardiology, Osaka General Medical Center, Osaka, Japan; 24 Department of Cardiology, Fujita Health University School of Medicine, Toyoake, Aichi Japan; 25 Department of Cardiology, Faculty of Medicine, University of Tsukuba, Tsukuba, Ibaraki Japan; 26 Department of Information and Computer Technology, Faculty of Engineering, Tokyo University of Science, Tokyo, Japan; 27 Department of Cardiovascular Medicine, Hanwa Memorial Hospital, 3-5-8 Minamisumiyoshi, Sumiyoshi-ku, Osaka, 558-0041 Japan; 28 The Osaka Medical Research Foundation for Intractable Diseases, Osaka, Japan

**Keywords:** Cardiovascular diseases, Heart failure, Hypertension

## Abstract

Characterized by ventricular and vascular stiffness, heart failure with preserved ejection fraction (HFpEF) has led to high morbidity and mortality. As azilsartan is an angiotensin receptor blocker with the highest myocardial and vascular affinities, azilsartan may improve the left ventricular (LV) diastolic function in patients with hypertension and either HFpEF or HF with mildly reduced ejection fraction (HFmrEF) more than candesartan. In this randomized, open-label trial, we randomly assigned 193 hypertensive patients with HF and LV ejection fraction ≥ 45% to 20 mg of azilsartan (n = 95) or 8 mg of candesartan (n = 98), once daily for 48 weeks. After the initiation of treatment, changes in the doses of the study drugs were permitted based on the patient’s conditions, including blood pressure (median dose at 48 weeks: azilsartan 20.0 mg/day, candesartan 8.0 mg/day). The primary endpoint was the baseline-adjusted change in the ratio of peak early diastolic transmitral flow velocity (E) to early diastolic mitral annular velocity (e′) (E/e′). Adjusted least-squares mean (LSM) change in E/e′ was − 0.8 (95% confidence interval [CI] − 1.49 to − 0.04) in the azilsartan group and 0.2 (95% CI − 0.49 to 0.94) in the candesartan group, providing the LSM differences of − 1.0 (95% CI − 2.01 to 0.03, P = 0.057). The median change in left atrial volume index was – 2.7 mL/m^2^ with azilsartan vs 1.4 mL/m^2^ with candesartan (P = 0.091). The frequency of adverse events related to hypotension and hyperkalemia did not differ between the groups. The current study did not provide strong evidence that azilsartan improves LV diastolic dysfunction, and further confirmatory study is required.

## Introduction

From the clinical point of view as well as a public health problem, heart failure (HF) is associated with remarkably high morbidity, mortality, and healthcare expenditures. Recently, both HF with preserved ejection fraction (HFpEF) and HF with mildly reduced EF (HFmrEF) have accounted for approximately one-third to one-half of the HF cases in epidemiological studies^1^. Left ventricular (LV) diastolic dysfunction is an essential component in either HFpEF or HFmrEF, which has been reported to result from myocardial and vascular disorders^2^. Elevation of either ventricular or vascular stiffness may collectively lead to an elevation in LV end-diastolic pressure, an ultimate marker for HFpEF, along with an afterload increase^3^. This finding raises a possibility that the decreases in vascular load, i.e. the capacitance’s increase or decreases of the systemic vascular resistance, can improve ventricular diastolic function^4,5^. Interestingly, the activation of renin–angiotensin aldosterone system (RAAS) induces ventricular and vascular remodeling^6^ associated with adverse clinical outcomes, and the inhibition of the RAAS should be one of the treatment options for HF; nonetheless, several clinical trials for HFpEF failed to exhibit the effects of RAAS inhibitors, including angiotensin II receptor blockers (ARBs). For example, the I-PRESERVE^7^ study showed that irbesartan had no effect on cardiovascular mortality and hospitalization in patients with HFpEF. However, a subanalysis of the I-PRESERVE trial exhibited the beneficial effects of irbesartan in HFpEF patients with levels of baseline plasma N-terminal pro-brain natriuretic peptide (NT-proBNP) below the median but not in patients with baseline levels above the median^8^, suggesting the effectiveness of irbesartan for certain types of HFpEF. The CHARM study showed that candesartan improved the clinical outcomes in HF patients with systolic dysfunction^9,10^ and provided a modest impact for the patients with left ventricular ejection fraction (LVEF) > 40%^11^, i.e. HFmrEF and HFpEF. Taken together with these previous results, ARBs with strong binding to angiotensin II type 1 receptors may elicit preventive effects on the progression of HFmrEF and HFpEF.

Azilsartan has a higher affinity for and slower dissociation from angiotensin II type 1 receptors than other ARBs^12^. Indeed, azilsartan exhibits 3.1 times higher affinity for arteries than candesartan even after a drug washout^13^. Our retrospective study showed that azilsartan improves LV diastolic dysfunction more than candesartan in patients with HF^14^, suggesting as well as inspiring us to prospectively evaluate azilsartan’s effects compared with candesartan on LV diastolic dysfunction.

## Results

### Study participants

**Figure 1:**
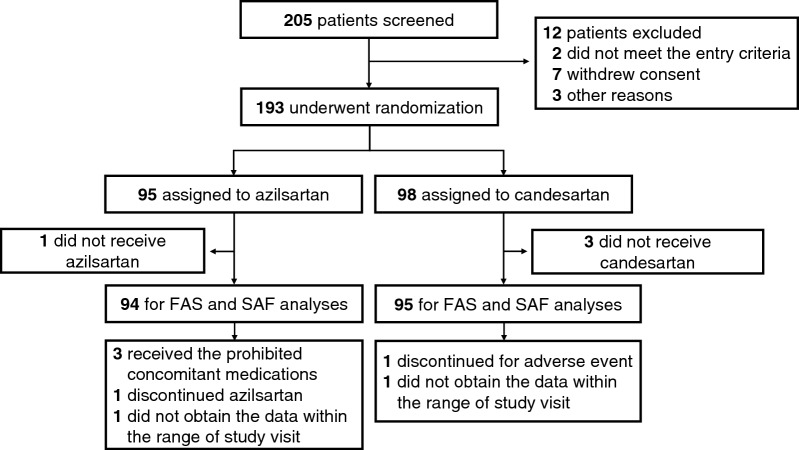
The study flow chart for the enrollment, randomization, and follow-up. The participants who did not receive a dose of either azilsartan or candesartan were excluded from the primary and safety analyses. Both FAS and SAF denotes full analysis set and safety analysis set, respectively.

Figure 1 shows the study flow chart for the enrolment, randomization, and follow-up. We screened 205 subjects from August 10, 2016 to June 18, 2019 at 23 institutions in Japan. Of these, 193 participants provided written informed consent and were randomized into 95 and 98 participants in the azilsartan and candesartan groups, respectively. No participants were lost during the follow-up. The participants treated with the study drug at least once (94 and 95 in the azilsartan and candesartan groups, respectively) were included in the full analysis and safety analysis sets. The follow-up was discontinued within 48 weeks in five and two patients in the azilsartan and candesartan groups, respectively.

The baseline characteristics of the patients in both groups shown in Table 1 were almost similar regarding gender, age, the peak early diastolic transmitral flow velocity (E) to the early diastolic mitral annular velocity (e′) (E/e′), principal cause of HF, presence of previous HF admission, hypertension, and diabetes mellitus, although estimated glomerular filtration rate (eGFR) and history of cerebral cardiovascular disease were higher (P = 0.028) and lower (P = 0.032) in the azilsartan group than in the candesartan group, respectively. The baseline echocardiographic data were also similar between the two groups (Tables S1 and S3). The percentage of HFpEF was 95.3% in the azilsartan group and 94.3% in the candesartan group (P = 0.746).

**Table 1:** Baseline characteristics.

	Azilsartan (N = 94)	Candesartan (N = 95)
Male (%)	50 (53.2)	58 (61.1)
Age (years)	75.0 (70.0 − 80.0)	76.0 (69.0 − 81.0)
BMI (kg/m^2^)	23.7 (21.5 − 26.1)	24.3 (22.0 − 26.6)
NYHA (I/II/III/IV), %	6.4/92.6/1.1/0	11.6/86.3/2.1/0
Smoking (current/past), %	3.2/43.6	9.5/45.3
Alcohol drinking (%)	44 (46.8)	55 (57.9)
eGFR (mL/min/1.73 m^2^)	57.1 (48.7 − 69.8)	53.0 (42.2 − 63.1)
E/e′	13.1 (10.5 − 15.7)	13.3 (10.2 − 16.1)
LVEF ≥ 50%, %	95.3	94.3
Previous HF admission (%)	23 (24.5)	27 (28.4)
Hypertension (%)	85 (90.4)	87 (91.6)
Diabetes mellitus (%)	40 (42.6)	38 (40.0)
Dyslipidemia (%)	66 (70.2)	63 (66.3)
Any cause of cancer (%)	15 (16.0)	16 (16.8)
COPD (%)	2 (2.1)	5 (5.3)
Kidney disease (%)	16 (17.0)	20 (21.1)
History of CVD (%)	17 (18.1)	30 (31.6)
Principal cause of HF-no. (%)		
Hypertensive	62 (66.0)	72 (75.8)
Ischemic	19 (20.2)	19 (20.0)
Valvular	8 (8.5)	2 (2.1)
Cardiomyopathy	4 (4.3)	1 (1.1)
Others	1 (1.1)	1 (1.1)
Device therapy-no. (%)		
Pacemaker	6 (6.4)	3 (3.2)
Defibrillator	1 (1.1)	0
CRT	0	0
Medications-no. (%)		
ACE inhibitor	4 (4.3)	2 (2.1)
ARB	53 (56.4)	49 (51.6)
Beta-blocker	56 (59.6)	60 (63.2)
Diuretic	32 (34.0)	37 (38.9)
MRA	12 (12.8)	13 (13.7)
Ca blocker	50 (53.2)	54 (56.8)
SGLT2 inhibitor	6 (6.4)	7 (7.4)

Data are shown as median and interquartile range (IQR; 25th and 75th percentile) or n (%).

*BMI* body mass index, *NYHA* New York Heart Association, *eGFR* estimated glomerular filtration rate, *LVEF* left ventricular ejection fraction, *HF* heart failure, *COPD* chronic obstructive pulmonary disease, *CVD* cerebral cardiovascular disease, *CRT* cardiac-resynchronization therapy, *ACE* angiotensin converting enzyme, *ARB* angiotensin-receptor blocker, *MRA* mineralocorticoid receptor antagonist, *SGLT2* sodium–glucose cotransporter 2.

### Administration of study drugs and follow-up

The median (interquartile range [IQR] [25th to 75th percentile]) durations of treatment with the study drug were 336.0 days (300.8–349.3 days) and 336.0 days (315.0–357.0 days) in the azilsartan and candesartan groups, respectively. The numbers of participants whose compliance rate was less than 80% or who suspended the treatment were 5 (5.3%) and 5 (5.3%) in the azilsartan and candesartan groups, respectively. The number of patients who were prescribed additional drugs for HF or hypertension was the same between the two groups (Table S4).

Of the 189 participants included in the full analysis set, the average E/e′ ratio using septal and lateral e′ at baseline and 48 weeks of the study analyzed at the core laboratory was obtained from 67 to 69 participants in the azilsartan and candesartan groups, respectively.

### Study outcomes

**Figure 2:**
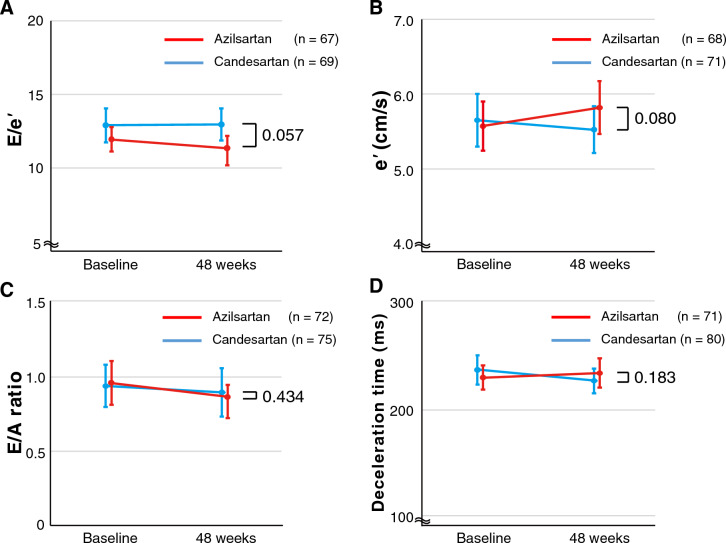
The changes in the parameters of left ventricular diastolic function with echocardiography from baseline to 48 weeks. (**A**) E/e′ ratio obtained from the averaged the values of septal and lateral e′, (**B**) e′ velocity obtained from the averaged values of septal and lateral e′, (**C**) E/A ratio and (**D**) the deceleration time of early diastolic mitral annular velocity. The statistical analyses of absolute changes in E/e′ from baseline to 48 weeks were performed using analysis of covariance adjusted for the baseline data. The absolute change in e′, E/A ratio and deceleration time from baseline to 48 weeks between the groups were compared using Student’s *t* test. Data are expressed as mean ± 95% confidence interval. E/e′ the ratio of peak early diastolic transmitral flow velocity (E) to early diastolic mitral annular velocity (e′), E/A ratio the ratio of peak early diastolic transmitral flow velocity (E) to atrial systolic transmitral flow velocity (**A**).

**Figure 3:**
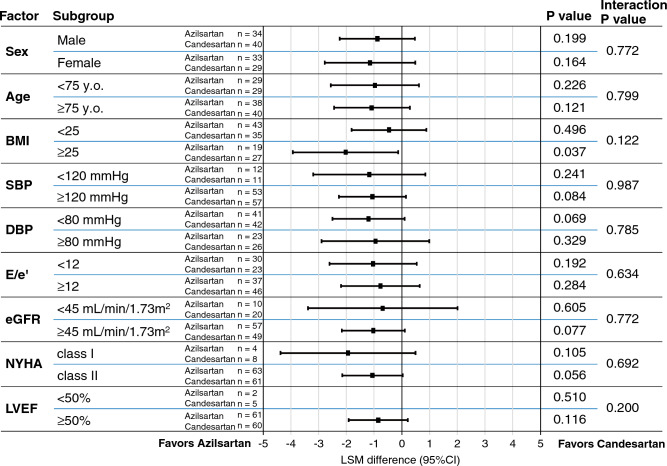
The changes in E/e′ from baseline to 48 weeks stratified by the potential influential factors. The statistical analyses of absolute changes in each parameter from baseline to 48 weeks were performed using analysis of covariance adjusted for the baseline data. LSM, 95% CI and p values were calculated from the analysis of covariance model. The LSM difference (3.0 ± 0.5, 95% CI − 8.34 to 14.21) in the patients with LVEF < 50% was omitted because the 95% CI was large due to small sample size. *BMI* body mass index, *SBP* systolic blood pressure, *DBP* diastolic blood pressure, E/e′ ratio the peak early diastolic transmitral flow velocity (E) to peak early diastolic mitral annulus velocity (e′), eGFR estimated glomerular filtration rate, *NYHA* New York Heart Association, *LVEF* left ventricular ejection fraction, *LSM* least-squares mean, *CI* confidence interval.

The primary endpoint of the baseline-adjusted change in E/e′ from the baseline to 48 weeks was decreased by azilsartan (the azilsartan group, least-squares mean [LSM]: − 0.8 ± 0.4, 95% CI  − 1.49 to − 0.04; the candesartan group, LSM: 0.2 ± 0.4, 95% CI  − 0.49 to 0.94; LSM difference,  − 1.0 ± 0.5, 95% CI  − 2.01 to 0.03, P = 0.057), although the difference was not statistically significant (Fig. 2A). The absolute change in e′ was not significantly different between the groups but tended to increase in the azilsartan group (Fig. 2B and Table S2). The absolute changes in E/A and mitral E wave deceleration time of other parameters relevant to diastolic function were similar in the two groups (Fig. 2C and D). The favorable trend of azilsartan on the primary endpoint was generally consistent across the prespecified subgroups (Fig. 3). Among the absolute changes in parameters of cardiac structure and LV systolic function provided by echocardiogram, the differences in LV end-diastolic diameter (LVDd) were greater in the azilsartan group than those in the candesartan group (P = 0.034) (Table S1). The absolute change of left atrial volume index (LAVi) was − 2.7 (IQR: − 6.4 to 2.6) mL/m^2^ in the azilsartan and 1.4 (IQR: − 5.8 to 7.0) mL/m^2^ in the candesartan groups, but was not statistically significant (P = 0.091) (Table S1). The absolute changes in LV end-systolic and end-diastolic elastance were not different between the azilsartan and candesartan groups (LV end-systolic elastance: the azilsartan group − 0.135 mmHg/mL vs. the candesartan group − 0.018 mmHg/mL, P = 0.499; LV end-diastolic elastance: the azilsartan group − 0.072 mmHg/mL vs. the candesartan group − 0.157 mmHg/mL, P = 0.584).

**Figure 4:**
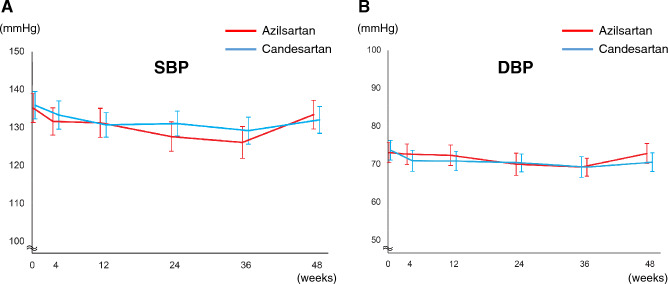
The changes in systolic (**A**) and diastolic (**B**) blood pressure at each study visit. Data are expressed as mean ± standard deviation. *SBP* systolic blood pressure, *DBP* diastolic blood pressure.

No significant differences were exhibited in either systolic blood pressure (BP), diastolic BP or heart rate between the two groups throughout the study (Fig. 4, Tables S5 and S6). The proportion of participants with the improvement of New York Heart Association (NYHA) functional class tended to be higher in the azilsartan group than in the candesartan group, although no statistical difference was observed (P = 0.235) (Fig. S2). The absolute changes in plasma NT-proBNP and aldosterone levels from baseline to 48 weeks were not different between the two groups (Table S7). Table S8 shows the results of cardiovascular events; there were no significant differences in the cardiovascular events in both groups. New onset of atrial fibrillation and flutter occurred in zero patients in the azilsartan group and two patients (2.1%) in the candesartan group.

### Safety

The safety analysis included all randomized patients who received at least one dose of the study drug (n = 94 and 95 in the azilsartan and candesartan groups, respectively) (Fig. 1). The incidences of adverse events were 64.9% and 48.4% in the azilsartan and candesartan groups (P = 0.033), respectively; the incidences of adverse events related to the study drugs were 12.8% and 16.8% in the azilsartan and candesartan groups, respectively (P = 0.559). The incidences of adverse drug reactions were 12.8 and 16.8% in the azilsartan and candesartan groups, respectively (P = 0.559). The incidences of serious adverse events in the azilsartan and candesartan groups were 20.2% and 11.6%, respectively (P = 0.154) (Table S9). The incidences of hypotension and hyperkalemia were 0% and 2.1% in the azilsartan group and 1.1% and 1.1% in the candesartan group, respectively. One patient each in the azilsartan and candesartan groups died; both were considered as non-drug-related death. No statistical difference was found for each adverse event. The summary of most commonly reported adverse events with an incidence greater than or equal to 2% is listed in Table S10.

## Discussion

In the present study, azilsartan showed a trend toward improvement in LV diastolic dysfunction in patients with either HFmrEF or HFpEF compared with candesartan, although the difference was not statistically significant (P = 0.057); azilsartan decreased LV E/e′ by 0.8, whereas candesartan increased it by 0.2 for 48 weeks, despite no significant differences in the time course of BP during 48 weeks between both groups. This result supports the hypothesis that the potential of azilsartan to improve LV diastolic dysfunction is superior to candesartan. Furthermore, the present study provided the potential evidence of the following: 1) e′, an index of LV relaxation speed increased by 0.1 cm/s in the azilsartan group and decreased by 0.2 m/s in the candesartan group (P = 0.080) and 2) azilsartan/candesartan decreased/increased LVDd (median 1.4 mm vs. 0.8 mm, p = 0.034), respectively. The most important implication of the present study is that azilsartan has the potential for improvement of LV dysfunction on the pathophysiology of HFmrEF or HFpEF.

The tissue Doppler index E/e′ is a routine parameter to assess LV diastolic function in patients with HFpEF in the 2021 European Society of Cardiology^15^ and the 2022 American College of Cardiology/American Heart Association/Heart Failure Society of America guidelines^16^, including other multistep echocardiographic assessments. Several clinical studies^17,18^ have identified E/e′ as an independent predictor of future cardiovascular events in patients with HFpEF; thus, E/e′ has been widely used as a surrogate endpoint in randomized control trials^19,20^.

However, recent studies suggest the limitation of E/e′ for assessing diastolic function in patients with HFpEF for (1) low sensitivity with LV filling pressure and (2) unreliability under certain situations. Further, global longitudinal strain and strain rate by speckle tracking correlates well with LV relaxation and filling pressure compared with E/e′^21,22^; therefore, speckle tracking analysis may provide a more accurate measure of changes in diastolic function. Large differences in strain data have been reported, depending on ultrasound vendors^23^. We did not unify the vendors in this study, so further study is needed. As mentioned above, E/e′ is unreliable in some situations, such as acute decompensated HF, having received cardiac-resynchronization therapy, history of mitral valve surgery and patients with severe mitral valve disease and annular calcification. Thereby, we excluded unreliable assessment in setting the exclusion criteria and by core laboratory analysis. We showed that azilsartan tended to improve not only E/e′ but also other echocardiographic parameters such as LAVi and estimated pulmonary artery systolic pressure, which supports our data that azilsartan may improve LV diastolic dysfunction.

The major issue in the present result is whether an improvement of E/e′ by − 1.0 in the azilsartan group compared with the candesartan group can provide a meaningful impact on cardiovascular events. Several reports showed the tight linkages between the changes in E/e′ and cardiovascular events. In HFmrEF or HFpEF patients with hypertension and patients with characteristics comparable to the present study, Zhou et al. showed that a unit rise of E/e′ is significantly associated with a 26% increase in cardiovascular events^24^. In HFpEF patients with hemodialysis, Iwabuchi showed a 7% significant increase per a unit rise of E/e′ in cardiovascular events^25^; in HFpEF patients enrolled in PARAGON-HF study, Shah showed a 4% significant increase in cardiovascular events^26^. In the HFpEF patients with non-valvular atrial fibrillation, a unit rise of E/e′ caused a 5% significant increase in cardiovascular events^27^. Taken together, a unit rise of E/e′ increases annual cardiovascular events from a few to a dozen percent in HFpEF patients, confirming the clinically significant impact of the present results. However, the present study did not show significant differences in cardiovascular events between azilsartan and candesartan groups. This disparity may be because the present study was not designed as an outcome study from the viewpoint of the length and population of the clinical study, and this study enrolled the relatively mild HFmrEF and HFpEF patients of NYHA I and II, wherein few cardiovascular events were expected to be observed during the echocardiogram 48 weeks following the entry.

The previous studies suggest that the pathophysiology of HFpEF is characterized by high ventricular and vascular stiffness^5,28^, and our hypothesis of the present study is attributable to the idea that the improvement of ventricular and vascular stiffness by therapy with a strong ARB may also improve the LV diastolic function. Myocardial property can be assessed by NT-proBNP; the plasma NT-proBNP levels are not different in both groups, suggesting that the improvement of E/e′ may not be attributable to the improvement of myocardial property caused by azilsartan although there is no evidence that a decrease in the plasma NT-proBNP levels is linked to an improvement of E/e′. Contrarily, several clinical studies suggest a robust relationship between the elevation in vascular load and worsening diastolic function^3,4^; Elzinga and Westerhof showed that the time to aortic peak flow decreases when aortic resistance increases or capacitance decreases along with the early onset of aortic valve opening^29^, and the early opening of the aortic valve and early timing of LV ejection slows LV relaxation^30^. This indicates that the changes in vascular compliance or resistance may alter the LV relaxation property via the changes in the LV ejection mode. However, the present study provided evidence that both azilsartan and candesartan decreased LV end-systolic/diastolic elastance to the same extent, suggesting that the difference in vascular effects between the azilsartan and candesartan groups does not explain the superiority of azilsartan for the improvement of LV diastolic dysfunction. Nevertheless, we cannot exclude the possibility that azilsartan changed the vascular compliance, which may improve the LV diastolic function.

There are several clinical trials using ARB in patients with HFpEF. The I-PRESERVE^7^ study using irbesartan, one of the ARBs, failed to demonstrate the beneficial effects of ARB on clinical outcomes in patients with HFpEF. The negative result is partially attributable to the small reduction of systolic BP by 3.8 mmHg in irbesartan^7^; however, in this study, there were no differences in the BP during the 48 weeks between the azilsartan and candesartan groups because the cardiologists mainly treated patients with hypertension and LV diastolic dysfunction in accordance with the Guideline for the Management of Hypertension published by Japanese Society of Hypertension in 2014^31^. The subanalysis of the I-PRESERVE study exhibited the beneficial effects of irbesartan in HFpEF patients with baseline NT-proBNP below the median but not in patients with levels above the median^8^. However, the effects of azilsartan were not influenced by the NYHA classification, i.e. the severity of HF. Contrarily, candesartan is a useful drug for HF worldwide^32^ as well as Japan. In the CHARM-Preserved study, in patients with an NYHA functional class II–IV chronic HF and LVEF higher than 40%, candesartan provides a considerable impact in preventing the admissions for covariate-adjusted HR = 0.86 [95% CI 0.74–1.00]^11^. The present study implies that azilsartan is superior to candesartan from the viewpoints of the effectiveness for LV diastolic dysfunction; therefore, some types of ARB, such as azilsartan, may be functionally or clinically effective for the patients with HFpEF.

The CHARM-Preserved study titrated to a target dose of candesartan as 32 mg (mean dose: 25.0 mg), but the J-TASTE trial administered 2 to 12 mg of candesartan (median dose at 48 weeks: 8.0 mg). The dose reactivity of candesartan in BP was observed up to 12 mg in Japanese patients, thus the approved maximum dose of candesartan is 12 mg in Japan. The ARCH-J study showed that 8 mg of candesartan reduced the worsening of HF by 14.8% in Japanese HF patients with LVEF ≤ 45%^33^. For these reasons, the maximum dose of candesartan was set as 12 mg (two subjects received 16 mg). As for azilsartan, no studies have been conducted in HF patients; therefore, the maximum dose was set as 40 mg, the approved maximum dose in Japan.

One may argue that the present study enrolled the patients with LVEF ≥ 45% because we were concerned that the patients with LVEF > 50% are not very numerous in Japan. Contrary to our expectation, only 5% of patients had LVEF < 50%, and the tendency of the superiority of azilsartan to candesartan is exhibited in Fig. 3; we believe that the present results are also the case in patients with HFpEF.

The incidence of total adverse events was higher in the azilsartan group than the candesartan group, but there were no differences in incidences of each adverse event, including hypotension and hyperkalemia, adverse drug reactions, severe adverse events and adverse events related to the study drugs. Therefore, azilsartan was generally tolerated but should be prescribed, considering the benefits and disadvantages obtained from the results of this study.

There are other limitations, including a small sample size and a relatively short follow-up, resulting in low statistical power. The J-TASTE trial was designed as an open-label trial, wherein participants and researchers were aware of their group allocation. To minimize the potential biases, the core lab sonographers performed the echocardiographic measurements, including the primary endpoint, in a blinded manner. Moreover, the judgment of the events was carried out by an independent committee according to prespecified criteria; therefore, we believe that our data provide clinically important implications. Although eGFR in the azilsartan group was slightly higher than that in the candesartan group, we did not consider the eGFR as a confounding factor because the effect of eGFR on the primary endpoint is small. We found no statistical significance regarding the additional drugs administered between the groups during the study period. Only a few patients received the additional drugs; therefore, this bias has little impact on our results. Finally, the sample size was set at 87 subjects per group without dropouts; however, E/e′ was obtained from 67 subjects in the azilsartan group and 69 in the candesartan group. This smaller number was mainly because septal and/or lateral e′ were not obtained by the core laboratory, as the images were not appropriate for analysis. Therefore, it is necessary to register the subjects whose images of echocardiogram are appropriate for measuring septal and lateral e′.

The strengths of the J-TASTE trial design include the multicenter design, central assessment system for echocardiography, suitable guidelines for control of BP by physicians, and assessments for novel biomarkers. In this study, the primary endpoint of the change in E/e′ from the baseline to 48 weeks was not statistically different between the azilsartan and candesartan groups. The current study did not provide the strong evidence that azilsartan improves LV diastolic dysfunction, and further confirmatory study is required.

## Methods

### Study design

Japan Working Group on the Effects of Angiotensin Receptor Blockers Selection (azilsartan vs. candesartan) on Diastolic Function in the Patients Suffering from Heart Failure with Preserved Ejection Fraction: J-TASTE trial was a multicenter, randomized, open-labeled (candesartan vs. azilsartan), and assessor(s)-blinded, active controlled using candesartan, parallel-group clinical trial, to test the hypothesis that azilsartan improves diastolic dysfunction in either HFmrEF or HFpEF patients with hypertension. This trial was first registered with the UMIN-CTR registry (UMIN000022556) on 31/05/2016 before the enrollment and also registered with the Japan Registry of Clinical Trials (jRCTs051180137) on 18/03/2019.

### Participants

Eligible requirements included an age of 20–85 years, previous diagnosis of hypertension, plasma BNP ≥ 40 pg/mL or NT-proBNP ≥ 125 pg/mL, the existence of a previous diagnosis of HF (present or prior history of HF hospitalization, or NYHA class ≥ II symptoms), E/e′ ≥ 8, and LVEF ≥ 45%. Major exclusion criteria were as follows: angiotensin converting enzyme inhibitor use at the consent, the presence of persistent atrial fibrillation, continuous lower systolic BP (< 90 mmHg), implanted mechanical ventricular device, patients waiting for heart transplantation and cardiac surgery, patients with moderate or severe valve disease, the history of mitral valve replacement or plasty, the history of constrictive pericarditis, diagnosed as hypertrophic cardiomyopathy, and an eGFR below 15 mL/min/1.73 m^2^. Full trial inclusion and exclusion criteria are documented in the protocol paper^34^.

### Ethics approval

The institutional review board of all participating centers and the Certified Review Board of Hyogo College of Medical approved this trial, and all patients provided written informed consent. This study was administered in accordance with the Declaration of Helsinki and the Clinical Trials Act.

### Randomization and masking

Allocation of patients to each treatment group (1:1) was administered after confirmation of the study’s enrollment by a covariate-adaptive randomization method (Pocock and Simon’s minimization method), including the following as considered factors: (1) age (≥ 75 or < 75 years), (2) E/e′ (E/e′ ≥ 15 or < 15), (3) eGFR (≥ 30 or < 30 mL/min/1.73 m^2^), and (4) history of hospitalization for HF (Yes or No). The investigators outsourced the randomization and allocation task to an independent contract research organization. Randomization and allocation were done automatically by the web system. Participants and their clinicians were not masked to the allocated treatment. The echocardiographic measurements, including the primary endpoint, were performed by the core lab sonographers in a blinded manner.

### Procedures

Administrative details of the study are described in the Fig. S1. The administration of the study drugs was started at either 20 mg of azilsartan once daily or 8 mg of candesartan once daily. The physicians could change the doses of the study drugs based on the patient’s conditions, including blood pressure [dose range: azilsartan 10–40 mg/day, candesartan 2–12 mg/day, median dose at 48 weeks: azilsartan 20.0 mg/day (10 mg 23.0%, 20 mg 55.2%, 40 mg 21.8%), candesartan 8.0 mg/day (2 mg 5.7%, 4 mg 18.2%, 6 mg 1.1%, 8 mg 56.8%, 12 mg 15.9%, 16 mg 2.3%)] and could also add other antihypertensive drugs, excluding other RAAS inhibitors, according to the Guideline for the Management of Hypertension published by Japanese Society of Hypertension in 2014^31^.

For the echocardiographic assessments, the same model of echocardiogram machine was utilized at the baseline and the end of the study for each patient; all data were obtained according to the standard operating procedure and sent to the core laboratory where all analyses were performed by the independent sonographers in a blinded manner. Ventricular dimensions were measured from either 2-D dimensional or M-mode images, according to the American Society of Echocardiography conventions. Left atrial volume and LVEF were determined by the biplane modified Simpson method. Relative wall thickness and LV mass index were calculated using the following formulae:

$${\text{Relative wall thickness }} = { 2 } \times {\text{ posterior wall thickness}}/{\text{LVDd}},$$

$${\text{LV mass }} = \, 0.{8 } \times \, \left[ {{1}.0{4 }\left( {{\text{end}} - {\text{diastolic interventricular septal thickness }} + {\text{ LVDd }} + {\text{ end}} - {\text{diastolic left ventricular posterior wall thickness}}} \right)^{{3}} - {\text{LVDd}}^{{3}} } \right] \, + \, 0.{6}.$$

The estimated pulmonary artery systolic pressure was calculated by the sum of the transtricuspid gradient and the estimated right atrial pressure^35^.

LV end-systolic and diastolic elastance were calculated using previously reported formulae^36,37^.

### Outcomes

While the change in E/e′ assessed by echocardiography from baseline to the end of the study (48 weeks) was the primary endpoint, the secondary endpoints included the changes in (1) e′; (2) E/atrial systolic transmitral flow velocity (A); (3) deceleration time of early diastolic mitral annular velocity; (4) LV and left atrial size; NYHA functional class; (5) NT-proBNP levels; (6) serum aldosterone levels; (7) BP at each visit; and (8) the incidence of cardiovascular events. E/e′ ratio and e′ were obtained by averaging the values of septal and lateral e′. Full secondary outcomes are documented in the protocol paper^34^. The judgment of the clinical events was carried out by an independent committee.

### Statistical analysis

Since this study is an exploratory study (i.e. is not a confirmatory study), the sample size was determined using precision-based method for the primary endpoint (change in E/e′ from the baseline to 48 weeks). If the sample size was set at 87 subjects per group, the difference in primary endpoint between the azilsartan and candesartan groups divided by the common standard deviation (i.e. standardized effect size) could be estimated with a sufficient precision which achieves the 0.6 width of the 95% confidence interval. Hence, the planned sample size was set at 95 subjects per group, with consideration for dropouts.

Analyses were performed based on the intention-to-treat principle. For the primary endpoint, i.e. the change from baseline to 48 weeks in E/e′, the analysis of covariance was used to calculate an adjusted mean difference and its 95% CI, where baseline E/e′ was included as a covariate. For subgroup analyses, regression methods were used with appropriate interaction terms (respective subgroup × treatment group). Secondary endpoints were descriptively analyzed, and while categorical variables were assessed using the Pearson chi-square test or Fisher’s exact test, continuous variables were analyzed with the Student’s *t* test. The paired differences between baseline and 48 weeks were analyzed using Wilcoxon signed rank sum test. All tests were two-sided, and a P value of < 0.05 was considered indicative of a statistically significant between-group difference. Statistical analysis software SAS, version 9.4 for Windows (SAS Institute, Cary, NC, USA) was utilized for all performed analyses. The statistical analysis plan was prepared separately and finalized before the database lock included more technical and detailed elaboration of the principal features stated in the protocol.

## Supplementary Information

Supplementary Information.

## Data Availability

The data analyzed in this study are available from the corresponding author upon reasonable request.

## Abbreviations

ACE: Angiotensin converting enzyme
ARBs: Angiotensin II receptor blockers
BMI: Body mass index
COPD: Chronic obstructive pulmonary disease
CRT: Cardiac-resynchronization therapy
CVD: Cerebral cardiovascular disease
DBP: Diastolic blood pressure
eGFR: Estimated glomerular filtration rate
HFmrEF: Heart failure with mildly reduced ejection fraction
HFpEF: Heart failure with preserved ejection fraction
LAVi: Left atrial volume index
LVDd: Left ventricular end-diastolic diameter
LVEF: Left ventricular ejection fraction
MRA: Mineralocorticoid receptor antagonist
NT-proBNP: N-terminal pro-brain natriuretic peptide
NYHA: New York Heart Association
RAAS: Renin–angiotensin aldosterone system
SBP: Systolic blood pressure
SGLT2: Sodium–glucose cotransporter 2
