# Supplementary material for: Efficacy of azilsartan on left ventricular diastolic dysfunction compared with candesartan: J-TASTE randomized controlled trial
Source: Sci Rep. 2023 Aug 2;13:12517. doi: 10.1038/s41598-023-39779-y (PMC10397297; doi:10.1038/s41598-023-39779-y)
Supplement: Supplementary file 1 — Supplementary Information. [file 41598_2023_39779_MOESM1_ESM.docx]

**Supplementary Information**

**Efficacy of azilsartan on left ventricular diastolic dysfunction compared with candesartan: J-TASTE randomized controlled trial**

Shin Ito, MD, PhD, Hiroyuki Takahama, MD, PhD, Masanori Asakura, MD, PhD, Yukio Abe, MD, PhD, Masayoshi Ajioka, MD, PhD, Toshihisa Anzai, MD, PhD, Takuo Arikawa, MD, PhD, Takaharu Hayashi, MD, PhD, Yorihiko Higashino, MD, Shinya Hiramitsu, MD, Noriaki Iwahashi, MD, PhD, Chisato Izumi, MD, PhD, Kazuo Kimura, MD, PhD, Koichiro Kinugawa, MD, PhD, Hidetaka Kioka, MD, PhD, Young-Jae Lim, MD, PhD, Ken Matsuoka, MD, PhD, Satoshi Matsuoka, MD, Hirohiko Motoki, MD, PhD, Sunao Nakamura, MD, PhD, Takafumi Nakayama, MD, PhD, Akihiro Nomura, MD, PhD, Taishi Sasaoka, MD, PhD, Shin Takiuchi, MD, PhD, Shigeru Toyoda, MD, PhD^8^, Tomoya Ueda, MD, PhD, Tetsuya Watanabe, MD, PhD, Akira Yamada, MD, PhD, Masayoshi Yamamoto, MD, PhD, Takashi Sozu, Ph, Masafumi Kitakaze, MD, PhD

**Table S1. Changes in echocardiographic parameters from baseline to 48 weeks**

|  | **Azilsartan (N = 94)** | | **Candesartan (N = 95)** | | **P value** |
| --- | --- | --- | --- | --- | --- |
| LVDd (mm) |  |  |  |  |  |
| Baseline | N = 90 | 48.2 (44.6–50.9) | N = 93 | 47.5 (44.4–50.2) | 0.592 |
| 48 weeks | N = 79 | 45.9 (43.1–50.1) | N = 88 | 48.3 (44.6–52.4) | 0.019 |
| Absolute change | N = 75 | −1.4 (−4.0–1.6) | N = 87 | 0.8 (−1.7–2.9) | 0.034 |
| LVDs (mm) |  |  |  |  |  |
| Baseline | N = 90 | 30.1 (25.4–33.3) | N = 93 | 29.7 (26.2–32.4) | 0.600 |
| 48 weeks | N = 79 | 27.6 (25.3–31.0) | N = 88 | 29.7 (26.6–33.3) | 0.043 |
| Absolute change | N = 75 | −0.4 (−3.4 to 2.4) | N = 87 | −0.7 (−2.4 to 2.9) | 0.659 |
| LAVi (mL/m^2^) |  |  |  |  |  |
| Baseline | N = 80 | 36.9 (30.7–43.0) | N = 78 | 35.3 (30.2–42.7) | 0.568 |
| 48 weeks | N = 71 | 33.8 (28.0–43.8) | N = 78 | 36.5 (30.0–44.5) | 0.338 |
| Absolute change | N = 66 | −2.7 (−6.4–2.6) | N = 72 | 1.4 (−5.8–7.0) | 0.091 |
| LV mass index (g/m^2^) |  |  |  |  |  |
| Baseline | N = 82 | 86.0 (72.2–100.2) | N = 83 | 83.0 (71.7–103.0) | 0.589 |
| 48 weeks | N = 75 | 82.4 (73.5–91.5) | N = 82 | 83.7 (71.2–101.4) | 0.317 |
| Absolute change | N = 70 | −2.9 (−10.5–10.0) | N = 81 | −2.0 (-12.4–5.2) | 0.964 |
| LVEF (%) |  |  |  |  |  |
| Baseline | N = 86 | 64.5 (59.0–69.0) | N = 87 | 63.0 (59.5–68.0) | 0.590 |
| 48 weeks | N = 75 | 64.0 (59.5–70.0) | N = 85 | 63.0 (58.0–68.0) | 0.096 |
| Absolute change | N = 69 | −1.0 (−6.0–5.0) | N = 78 | −1.0 (−6.0–4.0) | 0.749 |
| Estimated PASP (mmHg) |  |  |  |  |  |
| Baseline | N = 71 | 26.8 (23.0–32.2) | N = 57 | 27.3 (23.6–30.1) | 0.710 |
| 48 weeks | N = 61 | 25.5 (21.7–29.9) | N = 63 | 27.7 (23.7–31.4) | 0.113 |
| Absolute change | N = 50 | −1.8 (−8.0–3.1) | N = 43 | 0.3 (−3.6–5.0) | 0.082 |
| TR velocity (m/s) |  |  |  |  |  |
| Baseline | N = 84 | 2.33 (2.17–2.53) | N = 66 | 2.34 (2.23–2.53) | 0.712 |
| 48 weeks | N = 65 | 2.28 (2.08–2.51) | N = 73 | 2.34 (2.15–2.54) | 0.186 |
| Absolute change | N = 63 | −0.10 (−0.27–0.14) | N = 55 | 0.00 (−0.20–0.17) | 0.223 |

Data are expressed as the median and interquartile range (IQR; 25^th^ and 75^th^ percentile). The absolute changes in echocardiographic parameters from baseline to 48 weeks between the groups were compared using Student’s t-test. Abbreviations: LVDd, left ventricular end-diastolic diameter; LVDs, left ventricular end-systolic diameter; LAVi, left atrial volume indexed to body surface area; LVEF, left ventricular ejection fraction; PASP, pulmonary artery systolic pressure; TR, tricuspid regurgitation.

**Table S2. The paired differences in echocardiographic parameters between baseline and 48 weeks**

|  |  | **Azilsartan** |  |  | **Candesartan** |  |
| --- | --- | --- | --- | --- | --- | --- |
|  | N | Absolute change | P value | N | Absolute change | P value |
| **E/e′** | 67 | −0.7 (−2.6–1.7) | 0.142 | 68 | 0.0 (−2.1–2.6) | 0.917 |
| **e′ (cm/s)** | 68 | 0.1 (−0.6–1.2) | 0.216 | 70 | −0.2 (−0.8– 0.5) | 0.317 |
| **E/A** | 72 | −0.1 (−0.2–0.1) | 0.166 | 75 | 0.0 (−0.2–0.1) | 0.492 |
| **Deceleration time (ms)** | 71 | 4.0 (−37.5–47.5) | 0.649 | 80 | −6.2 (−48.8–27.7) | 0.193 |
| **LVDd (mm)** | 75 | −1.4 (−4.0–1.6) | 0.016 | 87 | 0.8 (−1.7–2.9) | 0.211 |
| **LVDs (mm)** | 75 | −0.4 (−3.4–2.4) | 0.481 | 87 | −0.7 (−2.4–2.9) | 0.891 |
| **LAVi (mL/m^2^)** | 66 | −2.7 (−6.4–2.6) | 0.042 | 72 | 1.4 (−5.8–7.0) | 0.462 |
| **LV mass index (g/m^2^)** | 70 | −2.9 (−10.5–10.0) | 0.495 | 81 | −2.0 (−12.4–5.2) | 0.246 |
| **LVEF (%)** | 69 | −1.0 (−6.0–5.0) | 0.591 | 78 | −1.0 (−6.0–4.0) | 0.412 |
| **Estimated PASP (mmHg)** | 50 | −1.8 (−8.0–3.1) | 0.051 | 43 | 0.3 (−3.6–5.0) | 0.638 |
| **TR velocity (m/s)** | 63 | −0.1 (−0.3–0.1) | 0.082 | 55 | 0.0 (−0.2–0.2) | 0.947 |

Data (absolute change) are expressed as the median and interquartile range (IQR; 25^th^ and 75^th^ percentile). The paired differences between baseline and 48 weeks were analyzed using Wilcoxon signed rank sum test. Abbreviations: E/e′, the ratio of peak early diastolic transmitral flow velocity (E) to early diastolic mitral annular velocity (e′); E/A, the ratio of peak early diastolic transmitral flow velocity (E) to atrial systolic transmitral flow velocity (A); Dct, the mitral E wave deceleration time; LVDd, left ventricular end-diastolic diameter; LVDs, left ventricular end-systolic diameter; LAVi, left atrial volume indexed to body surface area; LVEF, left ventricular ejection fraction; PASP, pulmonary artery systolic pressure; TR, tricuspid regurgitation.

**Table S3. Classification of LV geometry at the baseline**

|  | **Azilsartan** | **Candesartan** | **P value** |
| --- | --- | --- | --- |
| **Normal geometry**  RWT ≤0.42  LVMi ≤95 (♀) or ≤115 (♂) | N = 58 | N = 51 | 0.406 |
| **Concentric remodeling**  RWT >0.42  LVMi ≤95 (♀) or ≤115 (♂) | N = 7 | N = 13 |  |
| **Eccentric hypertrophy**  RWT ≤0.42  LVMi >95 (♀) or >115 (♂) | N = 15 | N = 15 |  |
| **Concentric hypertrophy**  RWT >0.42  LVMi >95 (♀) or >115 (♂) | N = 2 | N = 4 |  |

Differences between the groups were analyzed using the Pearson chi-square test.

Abbreviations: LV, left ventricle; RWT, relative wall thickness; LVMi; left ventricular mass index.

**Table S4. Additional drugs during the study between the groups**

|  | **Azilsartan (N = 94)** | **Candesartan (N = 95)** | **P value** |
| --- | --- | --- | --- |
| Medications-no. (%) | ·· | ·· |  |
| ACE inhibitor | 0 (0.0) | 0 (0.0) | 1.000 |
| ARB | ·· | ·· |  |
| Beta-blocker | 2 (2.1) | 3 (3.2) | 1.000 |
| Diuretic | 4 (4.3) | 5 (5.3) | 1.000 |
| MRA | 1 (1.1) | 0 (0.0) | 0.497 |
| Ca blocker | 2 (2.1) | 4 (4.2) | 0.682 |
| SGLT2 inhibitor | 1 (1.1) | 0 (0.0) | 0.497 |

Abbreviations: ACE, angiotensin converting enzyme; ARB, angiotensin-receptor blocker; MRA, mineralocorticoid receptor antagonist; SGLT2, sodium–glucose cotransporter 2.

**Table S5. Time course of blood pressure during 48 weeks between the groups**

|  | **Azilsartan (N = 94)** | | **Candesartan (N = 95)** | | **P value** |
| --- | --- | --- | --- | --- | --- |
| **Systolic blood pressure** | | | | | |
| Blood pressure level |  |  |  |  |  |
| Baseline | N = 92 | 134.0 (123.0–144.0) | N = 93 | 135.0 (126.0–147.0) | 0.773 |
| 4 weeks | N = 89 | 129.0 (119.0–145.0) | N = 89 | 132.0 (124.0–142.0) | 0.521 |
| 12 weeks | N = 90 | 130.5 (119.0–142.0) | N = 90 | 131.0 (120.0–140.0) | 0.833 |
| 24 weeks | N = 92 | 128.0 (117.0–140.0) | N = 93 | 129.0 (119.0–144.0) | 0.179 |
| 36 weeks | N = 80 | 127.5 (113.0–136.0) | N = 85 | 128.0 (117.0–139.0) | 0.261 |
| 48 weeks | N = 82 | 132.0 (123.0–142.0) | N = 92 | 131.5 (120.0–140.0) | 0.596 |
| Absolute change from baseline | | | | | |
| 4 weeks | N = 87 | −4.0 (−14.0–11.0) | N = 87 | −1.0 (−16.0–8.0) | 0.729 |
| 12 weeks | N = 88 | −3.0 (−13.0–8.5) | N = 89 | −4.0 (−20.0–8.0) | 0.365 |
| 24 weeks | N = 90 | −6.0 (−17.0–8.0) | N = 91 | −6.0 (−18.0–9.0) | 0.582 |
| 36 weeks | N = 78 | −8.5 (−23.0–1.0) | N = 83 | −7.0 (−21.0–6.0) | 0.277 |
| 48 weeks | N = 81 | −4.0 (−12.0–12.0) | N = 90 | −1.0 (−16.0–10.0) | 0.504 |
| **Diastolic blood pressure** | | | | | |
| Blood pressure level |  |  |  |  |  |
| Baseline | N = 91 | 72.0 (65.0–83.0) | N = 93 | 72.0 (64.0–83.0) | 0.738 |
| 4 weeks | N = 89 | 71.0 (64.0–81.0) | N = 89 | 71.0 (62.0–80.0) | 0.371 |
| 12 weeks | N = 90 | 70.0 (64.0–80.0) | N = 90 | 71.5 (62.0–79.0) | 0.417 |
| 24 weeks | N = 92 | 70.0 (60.0–80.0) | N = 93 | 70.0 (63.0–77.0) | 0.851 |
| 36 weeks | N = 80 | 70.0 (61.5–76.0) | N = 85 | 69.0 (61.0–78.0) | 0.983 |
| 48 weeks | N = 82 | 72.0 (66.0–81.0) | N = 92 | 70.0 (61.5–76.0) | 0.205 |
| Change from baseline | | | | | |
| 4 weeks | N = 86 | −2.0 (−8.0–6.0) | N = 87 | −2.0 (−10.0–4.0) | 0.288 |
| 12 weeks | N = 87 | −2.0 (−10.0–5.0) | N = 89 | 0.0 (−11.0–4.0) | 0.266 |
| 24 weeks | N = 89 | −4.0 (−10.0–2.0) | N = 91 | −4.0 (−15.0–6.0) | 0.857 |
| 36 weeks | N = 77 | −4.0 (−12.0–2.0) | N = 83 | −4.0 (−12.0–4.0) | 0.892 |
| 48 weeks | N = 80 | −2.0 (−7.5–5.0) | N = 90 | −4.0 (−11.0–5.0) | 0.305 |

Data are expressed as median and interquartile range (IQR; 25^th^ and 75^th^ percentile). The BP levels and absolute changes in BP from baseline between the groups were compared using Student’s t-test.

**Table S6. Time course of heart rate during 48 weeks between the groups**

|  | **Azilsartan (N = 94)** | | **Candesartan (N = 95)** | | **P value** |
| --- | --- | --- | --- | --- | --- |
| Heart rate, bpm | | | | | |
| Baseline | N = 88 | 69.0 (63.0–77.0) | N = 93 | 68.0 (60.5–76.0) | 0.297 |
| 4 weeks | N = 87 | 69.0 (60.0–75.0) | N = 89 | 62.0 (70.0–78.0) | 0.275 |
| 12 weeks | N = 89 | 70.0 (62.0–81.0) | N = 89 | 70.0 (62.0–76.0) | 0.664 |
| 24 weeks | N = 90 | 71.0 (61.5–76.0) | N = 93 | 70.0 (61.0–78.0) | 0.962 |
| 36 weeks | N = 78 | 68.0 (60.0–76.0) | N = 85 | 69.0 (62.5–77.5) | 0.702 |
| 48 weeks | N = 78 | 67.5 (60.0–78.0) | N = 90 | 67.5 (60.0–78.3) | 0.372 |
| Absolute change from baseline | | | | | |
| 4 weeks | N = 81 | 0.0 (−5.0–4.0) | N = 87 | 1.0 (−4.0–6.0) | 0.729 |
| 12 weeks | N = 84 | 0.0 (−5.0–6.8) | N = 88 | 1.0 (−4.0–7.0) | 0.974 |
| 24 weeks | N = 84 | 0.0 (−5.0–5.0) | N = 91 | 1.0 (−4.0–5.0) | 0.303 |
| 36 weeks | N = 73 | −2.0 (−5.0–5.0) | N = 83 | 0.0 (−7.0–7.0) | 0.559 |
| 48 weeks | N = 75 | −1.0 (−8.0–6.0) | N = 88 | 0.0 (−6.0–5.8) | 0.222 |

Data are expressed as median and interquartile range (IQR; 25^th^ and 75^th^ percentile). Heart rate and absolute changes in heart rate from baseline between the groups were compared using Student’s t-test.

**Table S7. Changes in laboratory data from baseline to 48 weeks**

|  |  | **Azilsartan (N = 94)** | | | **Candesartan (N = 95)** | | **P value** |
| --- | --- | --- | --- | --- | --- | --- | --- |
| **NT-proBNP, pg/mL** |  | |  |  | |  |  |
| Baseline | N=93 | | 235.0 (143.0–440.0) | N=93 | | 255.0 (152.5–515.5) | 0.128 |
| 48 weeks | N=82 | | 235.5 (130.5–453.3) | N=90 | | 203.0 (136.3–629.8) | 0.208 |
| Absolute change | N=82 | | 19.0 (−52.8 to 128.0) | N=88 | | −7.5 (−59.0 to 83.8) | 0.946 |
| **Aldosterone, pg/mL** |  | |  |  | |  |  |
| Baseline | N=93 | | 114.0 (79.8–151.0) | N=92 | | 119.5 (78.6–161.8) | 0.878 |
| 48 weeks | N=82 | | 105.5 (79.3–163.0) | N=89 | | 113.0 (81.3–160.0) | 0.844 |
| Absolute change | N=82 | | 0.1 (−31.9 to 24.6) | N=86 | | 5.2 (−37.2 to 35.0) | 0.624 |

Data are shown as median and interquartile range (IQR; 25^th^ and 75^th^ percentile). The absolute changes in NT-proBNP and Aldosterone from baseline to 48 weeks between the groups were compared using Student’s t-test.

Abbreviation: NT-proBNP, N-terminal pro-brain natriuretic peptide.

**Table S8. Efficacy outcomes**

|  | **Azilsartan**  **(N = 94)** | **Candesartan**  **(N = 95)** | **Hazard ratio**  **(95% CI)** | **P value** |
| --- | --- | --- | --- | --- |
| Composite endpoint  (Cardiovascular death or hospitalization for CVD) | 7 (7.4%) | 6 (6.3%) | 1.18 (0.41–3.38) | 0.782 |
| Composite endpoint  (Cardiovascular death or hospitalization for HF) | 3 (3.2%) | 3 (3.2%) | 1.01 (0.21–4.88) | 1.000 |
| Cardiovascular death | 1 (1.1%) | 0 (0.0%) | ·· | 0.497 |
| Death from any cause | 1 (1.1%) | 0 (0.0%) | ·· | 0.497 |
| Hospitalization for CVD | 7 (7.4%) | 6 (6.3%) | 1.18 (0.41–3.38) | 0.782 |
| Hospitalization for HF | 3 (3.2%) | 3 (3.2%) | 1.01 (0.21–4.88) | 1.000 |
| Additional therapy or dose escalation for HF caused by worsening of HF (24 weeks) | 1 (1.1%) | 2 (2.1%) | 0.51 (0.05–5.48) | 1.000 |
| Additional therapy or dose escalation for HF caused by worsening of HF (48 weeks) | 3 (3.2%) | 3 (3.2%) | 1.01 (0.21–4.88) | 1.000 |
| New onset of atrial fibrillation/flutter | 0 (0.0%) | 2 (2.1%) | ·· | 0.497 |

The hazard ratio and 95% CI were estimated by Cox proportional hazard model.

Abbreviations: CVD, cardiovascular disease; HF, heart failure; CI, confidence interval.

**Table S9. Summary of adverse events**

|  | **Azilsartan**  **(N = 94)** | **Candesartan**  **(N = 95)** | **P value** |
| --- | --- | --- | --- |
| **Adverse Events** |  |  |  |
| Total | 61 (64.9%) | 46 (48.4%) | 0.033 |
| Serious adverse events | 19 (20.2%) | 11 (11.6%) | 0.154 |
| Relationship |  |  |  |
| Unrelated | 49 (52.1%) | 30 (31.6%) | 0.007 |
| Related | 12 (12.8%) | 16 (16.8%) | 0.559 |
| Adverse Drug Reaction | 12 (12.8%) | 16 (16.8%) | 0.559 |

**Table S10. Summary of most commonly reported adverse events with an incidence ≥2%**

|  | **Azilsartan**  **(N = 94)** | **Candesartan**  **(N = 95)** | **P value** |
| --- | --- | --- | --- |
| Cardiac disorders | 8 (8.5%) | 8 (8.4%) | 1.000 |
| Angina pectoris | 2 (2.1%) | 0 (0.0%) | 0.246 |
| Cardiac failure congestive | 2 (2.1%) | 1 (1.1%) | 0.621 |
| Cardiac failure | 1 (1.1%) | 4 (4.2%) | 0.368 |
| Atrial fibrillation | 1 (1.1%) | 2 (1.1%) | 1.000 |
| Palpitation | 0 (0.0%) | 2 (2.1%) | 0.497 |
| Gastrointestinal disorders | 11 (11.7%) | 8 (8.4%) | 0.611 |
| Constipation | 3 (3.2%) | 2 (2.1%) | 0.682 |
| Abdominal discomfort | 2 (2.1%) | 0 (0.0%) | 0.246 |
| General disorders and administration site conditions | 8 (8.5%) | 4 (4.2%) | 0.250 |
| Chest discomfort | 3 (3.2%) | 0 (0.0%) | 0.121 |
| Peripheral edema | 2 (2.1%) | 1 (1.1%) | 0.621 |
| Neoplasms benign, malignant, and unspecified | 6 (6.4%) | 3 (3.2%） | 0.330 |
| Infections and infestations | 13 (13.8%） | 12 (12.6%) | 0.977 |
| Nasopharyngitis | 4 (4.3%) | 9 (9.5%) | 0.250 |
| Gastroenteritis | 2 (2.1%) | 1 (1.1%) | 0.621 |
| Urinary tract infection | 2 (2.1%) | 1 (1.1%) | 0.621 |
| Injury, poisoning, and procedural complications | 8 (7.4%) | 4 (4.2%) | 0.250 |
| Investigations | 11 (11.7%) | 9 (9.5%) | 0.794 |
| Blood potassium increased | 2 (2.1%) | 1 (1.1%) | 0.621 |
| Blood pressure increased | 6 (6.4%) | 5 (5.3%) | 0.986 |
| Blood uric acid increased | 2 (2.1%) | 2 (2.1%) | 1.000 |
| C-reactive protein increased | 2 (2.1%) | 4 (4.2%) | 0.682 |
| Blood creatine phosphokinase increased | 1 (1.1%) | 2 (2.1%) | 1.000 |
| Metabolism and nutrition disorders | 10 (10.6%) | 4 (4.2%) | 0.104 |
| Dehydration | 2 (2.1%) | 0 (0.0%) | 0.246 |
| Hyperkalemia | 2 (2.1%) | 1 (1.1%) | 0.621 |
| Musculoskeletal and connective tissue disorders | 6 (6.4%) | 5 (5.3%) | 0.986 |
| Arthralgia | 2 (2.1%) | 0 (0.0%) | 0.246 |
| Back pain | 2 (2.1%) | 0 (0.0%) | 0.246 |
| Myalgia | 0 (0.0%) | 2 (2.1%) | 0.497 |
| Immune system disorders | 1 (1.1%) | 2 (2.1%) | 1.000 |
| Seasonal allergy | 1 (1.1%) | 2 (2.1%) | 1.000 |
| Nervous system disorders | 10 (10.6%) | 8 (8.4%) | 0.786 |
| Cerebral infarction | 2 (2.1%) | 0 (0.0%) | 0.246 |
| Hypoesthesia | 2 (2.1%) | 0 (0.0%) | 0.246 |
| Dizziness | 1 (1.1%) | 5 (5.3%) | 0.211 |
| Psychiatric disorders | 4 (4.3%) | 1 (1.1%) | 0.211 |
| Insomnia | 3 (3.2%) | 1 (1.1%) | 0.368 |
| Renal and urinary disorders | 2 (2.1%) | 8 (8.4%) | 0.100 |
| Nocturia | 1 (1.1%) | 2 (2.1%) | 1.000 |
| Renal impairment | 1 (1.1%) | 3 (3.2%) | 0.621 |
| Chronic kidney disease | 0 (0.0%) | 2 (2.1%) | 0.497 |
| Respiratory, thoracic, and mediastinal disorders | 4 (4.3%) | 3 (3.2%) | 0.721 |
| Skin and subcutaneous tissue disorders | 7 (7.4%) | 8 (8.4%) | 1.000 |
| Vascular disorders | 2 (2.1%) | 3 (3.2%) | 1.000 |
| Ear and labyrinth disorders | 2 (2.1%) | 0 (0.0%) | 0.246 |

Adverse events were coded by the MedDRA/J version 23.1.


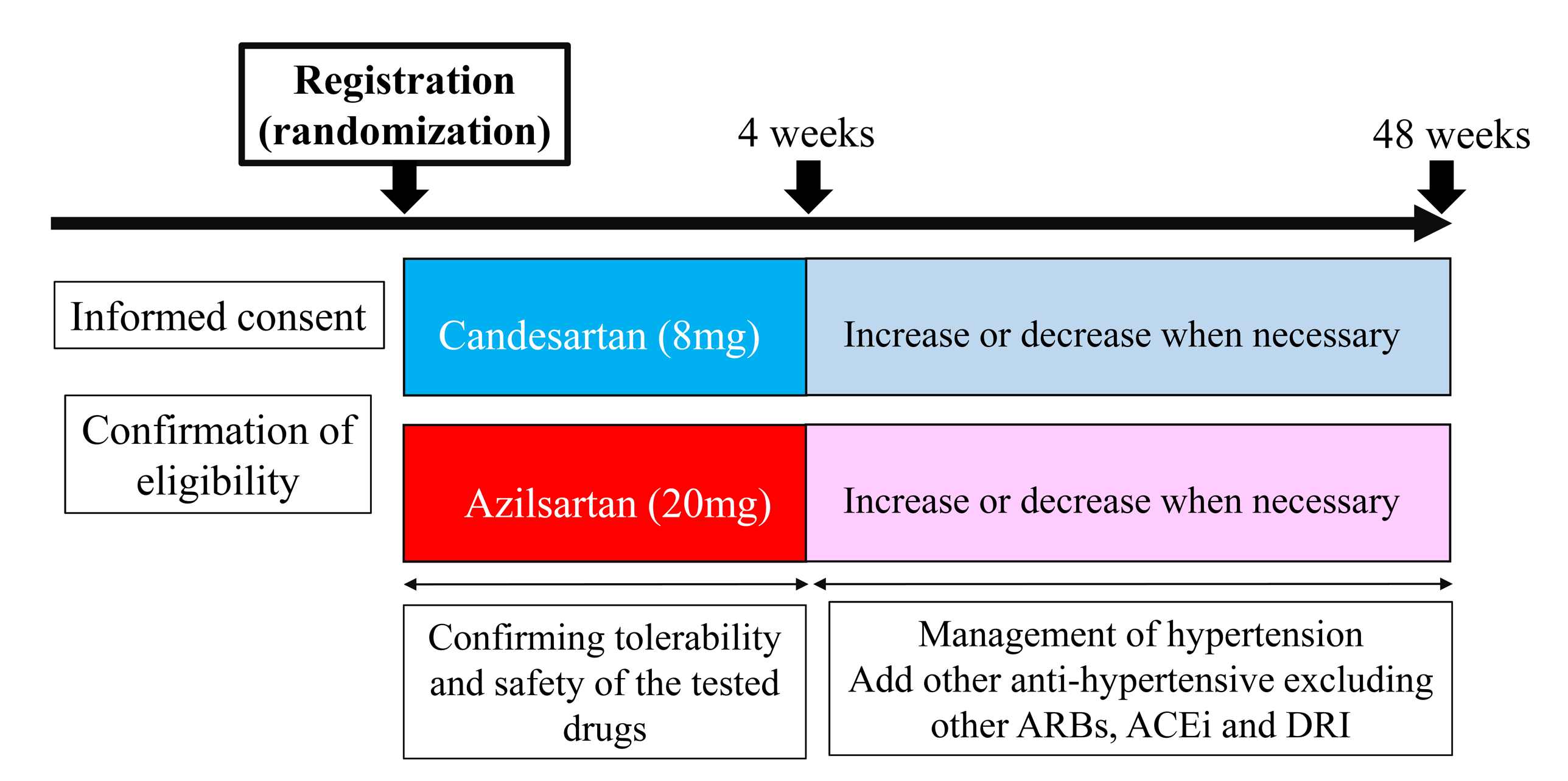


**Figure S1. Study protocol of the present study**

Abbreviations: ARB, angiotensin II receptor blocker; ACEi, angiotensin converting enzyme inhibitor; DRI, direct renin inhibitor.


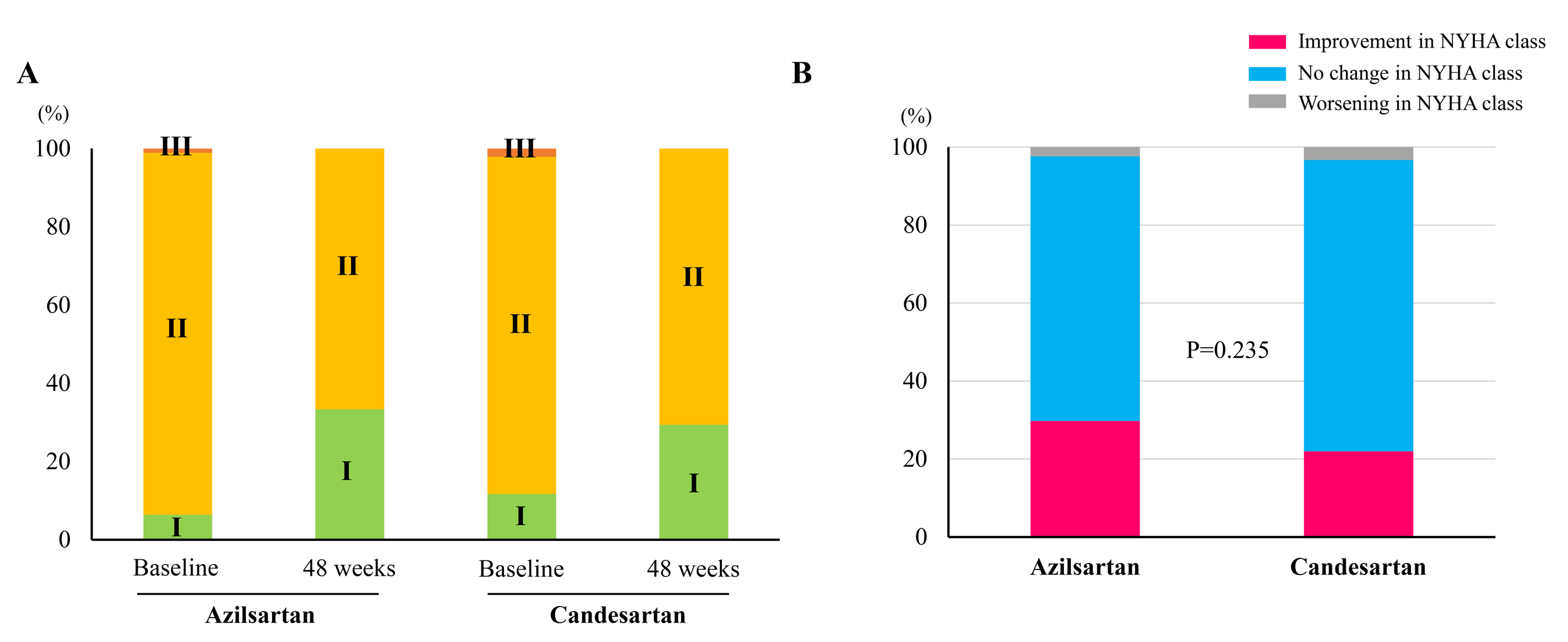


**Figure S2. The changes in NYHA functional class from baseline to 48 weeks.**

A: the proportion of NYHA functional class, B: the changes in NYHA functional class after 48 weeks. The statistical analyses of changes in NYHA functional class were performed using the Cochran–Mantel–Haenszel test.

Abbreviations: NYHA, New York Heart Association.
